# Apples and Cardiovascular Health—Is the Gut Microbiota a Core Consideration?

**DOI:** 10.3390/nu7063959

**Published:** 2015-05-26

**Authors:** Athanasios Koutsos, Kieran M. Tuohy, Julie A. Lovegrove

**Affiliations:** 1 Hugh Sinclair Unit of Human Nutrition and Institute for Cardiovascular and Metabolic Research (ICMR), Department of Food and Nutritional Sciences, University of Reading, Reading RG6 6AP, UK; E-Mail: a.koutsos@pgr.reading.ac.uk; 2 Nutrition and Nutrigenomics Group, Department of Food Quality and Nutrition, Research and Innovation Centre, Fondazione Edmund Mach, San Michele all’Adige, Trento 38010, Italy; E-Mail: kieran.tuohy@fmach.it

**Keywords:** apples, juice, fiber, pectin, polyphenols, cardiovascular, gut microbiota, blood lipid, cholesterol, vascular, inflammation

## Abstract

There is now considerable scientific evidence that a diet rich in fruits and vegetables can improve human health and protect against chronic diseases. However, it is not clear whether different fruits and vegetables have distinct beneficial effects. Apples are among the most frequently consumed fruits and a rich source of polyphenols and fiber. A major proportion of the bioactive components in apples, including the high molecular weight polyphenols, escape absorption in the upper gastrointestinal tract and reach the large intestine relatively intact. There, they can be converted by the colonic microbiota to bioavailable and biologically active compounds with systemic effects, in addition to modulating microbial composition. Epidemiological studies have identified associations between frequent apple consumption and reduced risk of chronic diseases such as cardiovascular disease. Human and animal intervention studies demonstrate beneficial effects on lipid metabolism, vascular function and inflammation but only a few studies have attempted to link these mechanistically with the gut microbiota. This review will focus on the reciprocal interaction between apple components and the gut microbiota, the potential link to cardiovascular health and the possible mechanisms of action.

## 1. Introduction

A high intake of plant derived foods such as fruits, vegetables and whole grains can have a preventative effect against cardiovascular diseases (CVD) [1,2,3]. The mechanisms are not entirely clear but plant polyphenols and fiber are considered the principal mediators. Apples are among the most popular and frequently consumed fruits in the world, because of their availability throughout the year, in a variety of forms including fresh fruit, juice, cider, concentrate and puree [4], and the general perception that apples are good for health [5]. Epidemiological studies support the view that frequent apple consumption is associated with a reduced risk of chronic pathologies such as cardiovascular disease, specific cancers, and diabetes [6,7,8,9,10]. Data from intervention studies in humans and animals suggest that apple intake may positively affect lipid metabolism [11,12,13,14,15], weight management [16], vascular function [17,18] and inflammation [19,20,21,22]. Apples are a rich source of polyphenols and fiber. The main polyphenol classes in increasing order are: Dihydrochalcones, flavonols, hydroxycinnamates and flavanols (catechin and proanthocyanidins (PAs)) [23]. Readily absorbed polyphenols such as flavanol monomers may be responsible for some of the health effects. However, larger polyphenol molecules such as the PAs, a major class of apple polyphenols, together with pectin, the main soluble fiber in apples and other cell wall components, reach the colon and undergo extensive bioconversion by colonic microbiota producing metabolites that may have local intestinal effects whilst in the gut, and systemic effects after absorption. Apple polyphenols and fiber may also beneficially modulate the gut microbiota composition and activity [24,25,26,27]. The gut microbiota may serve as a potential novel target for the prevention of CVD [28].

In this review we will present the major apple components and their reciprocal interaction with the gut microbiota; the evidence relating to the ability of apples to reduce CVD risk from *in vitro* and *in vivo* studies in animals and humans focusing on lipid metabolism, vascular function, blood pressure and inflammation; and finally the possible mechanisms which link the human gut microbiota and gut function to reduced risk. 

## 2. Apple Components

Apples are low in fat and high in carbohydrate, with fructose as the predominant sugar. Apples are also a rich source of vitamins (mainly C and E), minerals (potassium and magnesium), triterpenoids, such as ursolic acid, fiber (soluble and insoluble) and polyphenols (Table 1). 

**Table 1:** Composition of apples (*Malus domestica*), raw with skin. USDA National Nutrient Database for Standard Reference.

Components	Value per 100 g
Energy (kcal)	52
Energy (kJ)	218
Water (g)	85.56
Total carbohydrates (g)	13.81
^§^ Total dietary fiber (g)	2.21
^§^ Insoluble fiber (g)	1.54
^§^ Soluble fiber (g)	0.67
Total sugars (g)	10.39
Fructose (g)	5.9
Glucose (dextrose) (g)	2.43
Sucrose (g)	2.07
Starch (g)	0.05
Protein (g)	0.26
Total lipid (fat) (g)	0.17
Fatty acids, total polyunsaturated (g)	0.051
Fatty acids, total monounsaturated (g)	0.007
Fatty acids, total saturated (g)	0.028
Vitamin C, total ascorbic acid (mg)	4.6
Thiamin (mg)	0.017
Riboflavin (mg)	0.026
Niacin (mg)	0.091
Vitamin B-6 (mg)	0.041
Folate, DFE (μg)	3
Vitamin A, RAE (μg)	3
Vitamin A, IU (IU)	54
Vitamin E (alpha-tocopherol) (mg)	0.18
Vitamin K (phylloquinone) (μg)	2.2
Calcium, Ca (mg)	6
Iron, Fe (mg)	0.12
Magnesium, Mg (mg)	5
Phosphorus, P (mg)	11
Potassium, K (mg)	107
Sodium, Na (mg)	1
Zinc, Zn (mg)	0.04
* Total polyphenols (mg)	110.20
* Flavanols (mg)	96.33
* Hydroxycinnamates (mg)	14.21
* Flavonols (mg)	5.66
* Dihydrochalcones (mg)	4.17
^#^ Anthocyanins (mg)	1.62

^§^ Data from Li *et al.*, 2002 [29]; * data from Vrhovsek *et al.*, 2004 [23]; ^#^ only in red apples.

### 2.1. Apple Fiber

Apples contain approximately 2%–3% fiber [29,30]. They are rich in insoluble fiber, including cellulose and hemicullose, with pectin as the major soluble fiber containing homogalacturonans (consisting of long chains of α-(1–4) linked galacturonic acids) and rhamnogalacturonans. The degree of methylation, DM (or degree of esterification), of the galacturonic acid residues strongly influence the functional and physicochemical properties [31,32,33]. It has been reported that apple pectin has cholesterol lowering properties [34] and beneficial effects on glucose metabolism [35]. The structural parameters of pectin, including molecular weight and DM, have a major influence on the degree of these effects [32,34,36,37]. Moreover, pectin is a gelling agent and impacts on transit time, gastric emptying and nutrient absorption from the gut [34,38,39,40,41]. It is resistant to degradation by gastric acid and intestinal enzymes and thus, reaches the colon where it is fermented by the gut microbiota into short chain fatty acids (SCFA) [26,42,43,44]. 

### 2.2. Apple Polyphenols 

The polyphenol content of apples has been extensively measured in various studies [4,23,45,46,47] with only the most recent including the determination of oligomeric and polymeric PAs (representing approximately 80% of apple polyphenols) [4,23]. PAs, also known as condensed tannins, are oligomers and polymers of flavanols and are composed mainly of (−)-epicatechin units, although some of the terminal units may be from (+)-catechin. The most common subclass of PAs is the procyanidins (PCs) consisting of (epi)catechin units [48]. Vrhovsek *et al.* (2004) reported the polyphenol content in apples representing 8 of the most widely cultivated varieties in south and west Europe [23]. The mean concentration of total polyphenols was 110.2 mg/100 g of fresh fruit and ranged from 66.2 mg to 211.9 mg/100 g according to the following increasing order: Fuji, Braeburn, Royal Gala, Golden Delicious, Morgenduft, Granny Smith, Red Delicious and Renetta [23]. Flavanols (catechin and PAs) was the major class of apple polyphenols (71%–90%), followed by hydroxycinnamates (4%–18%), flavonols (1%–11%), dihydrochalcones (2%–6%) and anthocyanins (1%–3%), only in red apples [23]. Similarly a later study by Wojdylo *et al.* (2008) on 67 old and new varieties grown in Western Europe reported, flavanols (catechin and oligomeric PCs), 80%, hydroxycinnamic acids, 1%–31%, flavonols, 2%–10%, dihydrochalcones, 0.5%–5% and in red apples, anthocyanins, 1% [4]. The polyphenol profiles among several varieties are similar, but the concentration range varies [45,49,50,51,52] due to differences in cultivars, growing conditions (light availability), maturity, storage, extraction procedures, analytical techniques and pre- or post-harvest factors [47]. 

Phenolic compounds in apples are not evenly distributed in the fruit tissue. Despite the small contribution of apple peel (6%–8%) to whole fruit weight [53], peel contains a significantly higher content of phenolics. In particular all the flavonols (quercetin conjugates) and anthocyanins, as well as an important part of dihydrochalcones (phloridzin and phloretin glycosides) [23,46]. Moreover, the peel contains large amounts of PCs, (−)-epicatechin, (+)-catechin which also appear in the flesh but in lower concentrations [54]. Flesh has higher chlorogenic and some dihydrochoalcones [54]. The high content of polyphenols in the peel has been attributed to their defensive role against pathogens which may protect the fruit [55]. Apple juice contains only small amounts of quercetin glycosides and dihydrochalcones. Moreover, the technological processing is crucial. Clear apple juice has a small polyphenol content due to the oxidative conditions during the pulping and pressing and the final clarification process [56]. Cloudy apple juice on the other hand may maintain an important polyphenol amount due to anaerobic conditions and the lack of the clarification step [56,57].

#### 2.2.1. Absorption and Bioavailability of Apple Polyphenols

The health effects of polyphenols are likely to depend upon the initial dose, absorption and bioavailability [58]. The absorption of polyphenols is influenced by various factors such as their physicochemical properties (e.g., molecular size, degree of polymerization (DP), solubility, pKa), biological factors (through their passage in the gastrointestinal tract), food matrix, interaction with other dietary components and gut microbiota composition [59,60]. Aglycones and a few glycosides, mainly the low molecular weight polyphenols, can be directly absorbed in the small intestine [61]. High molecular weight polyphenols such as polymeric PAs reach the colon almost unchanged where they are transformed by the intense metabolic activity of the gut microbiota, a requirement for their absorption [61]. Glycosylated polyphenols require loss of their sugar moiety and esterified phenolic acids are hydrolyzed. In the brush borders of the intestine and after absorption in the liver, polyphenols undergo phase II metabolism leading to the formation of glucuronides, sulphates or methylated derivatives [61]. An increasing evidence base indicates that the concentration of parent polyphenols in human plasma is lower, sometimes by several orders of magnitude, than the concentration of pure polyphenols or plant extracts observed to have potential health effects *in vitro*. [59,61]. Moreover, bioconverted and conjugated forms of intact polyphenols could have higher biological activity than their parent compounds [61]. Absorbed polyphenols can either circulate in the blood reaching the target tissues or be resecreted into the intestine via the bile as a result of the enterohepatic circulation. The latter fraction can be either deconjugated by gut microbes and absorbed, or further metabolized by the gut microbiota [59,61,62]. A detailed review of the bioavailability and metabolism of the apple derived polyphenols in humans is presented by Bergmann *et al.* [63]. 

PCs are the major polyphenols in apples and cloudy apple juice, with only the monomeric, and to a lesser extent dimeric fractions, readily absorbed [64,65]. Oligomeric PCs may be cleaved into smaller units during their passage through the gastrointestinal tract, however, the major fraction (90%) is not absorbed and thus, reaches the colon [66]. Both *in vitro* and animal studies support the theory that a high polymerization decreases intestinal absorption [67]. Fifty-eight percent of polyphenols present in cloudy apple juice were absorbed or degraded, with the remaining detected in the ileostomy fluid [66]. 

Hydroxycinnamic acids are also an important group of polyphenols in apple products. In a study by Hagl *et al.* (2011), 10 healthy ileostomy subjects consumed 0.7 L of apple smoothie containing 60% of cloudy apple juice and 40% apple puree [60]. After 8 hours, 63% of the total polyphenols and d-(–)-quinic acid were found in the ileostomy bags compared with 60.9% of the hydroxycinnamic acids [60] with only 28.1% of hydroxycinnamic acids from pure cloudy apple juice [66]. Differences in the amount consumed and the quantity of cell wall compounds in apple smoothie may be responsible for the inconsistent results [60,66]. Hydroxycinnamic acids, including caffeoylquinic acids and *p*-coumaroylquinic acids, may be metabolized or hydrolyzed in the small intestine leading to d-(−)-quinic acid and caffeic or *p*-coumaric liberation which can be absorbed [60,66]. However, a significant proportion of chlorogenic acid (5-caffeoylquinic acid), may reach the colon where it can be further metabolized by the gut microbiota [59,60,67,68]. 

It has been reported that quercetin from apples is less bioavailable than that found in onions, due to differences in the quercetin conjugates present [69], suggesting that the sugar moiety affects absorption. [67]. Quercetin aglycones and glucosides, present in onions, are more bioavailable compared to quercetin monoglycosides and quercetin rutinosides found in apples [69,70]. Other studies reported that quercetin glucosides were more effectively absorbed compared to pure quercetin [67]. The main quercetin glycosides detected in human ileostomy bags after the consumption of apple smoothie or cloudy apple juice were 3-*O*-rhamnoside and 3-*O*-arabinoside, with higher recovery rates after apple smoothie consumption (46.3%) [60] compared with the cloudy apple juice (2.9%) [66]. In the large intestine, microorganisms can cleave the glycosidic bonds and free quercetin which can be absorbed or further metabolized by the gut microbiota [71]. 

The main dihydrochalcones include phloretin 2′-*O*-glucoside and phloretin-2′-*O*-(2′′-*O*-xylosyl) glucoside and are characteristic ingredients of apples and apple products. In a human study by Marks *et al.* (2009) the absorption and metabolism of the major apple dihydrochalones were explored in 9 healthy and 5 ileostomy subjects after the consumption of 500 ml of apple cider [72]. Phloretin glycosides were metabolized to phloretin 2´-*O*-glucuronide in both the healthy and ileostomy volunteers. Moreover, phloretin-2′-*O*-(2′′-*O*-xylosyl) glucoside was present in the ileal fluid together with the aglycone phloretin, two phloretin-*O*-glucuronides, and two phloretin-*O*-sulfates, but none of the ingested phloretin 2′-*O*-glucoside [72]. In total, 38.6% of the total dihydrochalcones were detected in the ileal fluid. These findings are consistent with Kahle *et al.* (2007) and Hagl *et al.* (2011) [60,66]. 

## 3. The Human Gut Microbiota—Effects of Fiber and Polyphenols 

The human gut is host to a diverse collection of microorganisms, comprising approximately 10^12^ microbial cells per gram of gut content and up to 1000 different species [73,74]. This collection of microorganisms is called the gut microbiota. The human colon is dominated mainly by two phyla, the *Firmicutes* and *Bacteroidetes*, representing more than 90% of all the phylotypes, followed by lower relative abundances of Actinobacteria, Proteobacteria, Fusobacteria and Verrucomicrobia [75]. The gut microbiota plays an important role in human health by increasing the efficiency of ‘energy harvest’ from the diet through the metabolism of nondigestible dietary components, maintaining immune homeostasis, synthesizing vitamins, such as B_12_ and K, providing a barrier to invading pathogenic microorganisms and reinforcing the intestinal epithelial cell tight junctions [76]. Bifidobacteria, lactobacilli and butyrate producing bacteria, such as *Faecalibacterium prausnitzii and Eubacterium rectale*, are commonly considered as health promoting bacteria involved in saccharolytic fermentation producing SCFA [75,76]. In contrast the overgrowth of other bacteria, such as the *Enterobacteriaceae* and certain clostridia groups, is associated with negative health implications. Aberrant gut microbiota composition has been associated with metabolic diseases including obesity, type I and type II diabetes and atherosclerosis, certain cancers and autoimmune diseases [77,78,79]. It has been shown that obese people have an increase ratio of *Firmicutes*/*Bacteroidetes* compared to lean subjects [80], however, this was not confirmed by later studies [81,82]. More recently it has been suggested that differences in microbiota diversity may be more important, in these pathologies, than changes in specific bacteria populations [83].

Gut microbiota profiles can be modified through diet [79,80,84]. The type and quantity of food components that reach the colon have an important impact on microbiota composition and activity [85]. Dietary fiber, a major substrate for colonic fermentation, plays an important role against the development of chronic diseases such as type 2 diabetes, heart disease, obesity and cancers [86,87,88,89]. This is particularly true for prebiotic fiber, a term first introduced in the mid-1990s referring to a class of fiber that may beneficially alter the colonic microbiota [90]. According to the recent definition by Gibson *et al.* (2010), a dietary prebiotic is ‘‘a selectively fermented ingredient that results in specific changes in the composition and/or activity of the gastrointestinal microbiota, thus conferring benefit(s) to host health’’ [91]. Well established prebiotics include inulin, fructooligosaccharides, galactooligosaccharides and lactulose, which have been consistently reported to increase relative numbers of bifidobacteria and lactobacilli [91]. Moreover, fiber is the main energy source for the gut microbiota leading to production of SCFAs butyrate, acetate and propionate [85]. These organic acids have multiple functions in the host, as key energy sources for intestinal mucosa, liver, muscle or other peripheral tissues contributing about 7%–8% of daily energy requirements. But more importantly they play a significant role in cell function, immune system, lipid metabolism, gut motility and permeability, affecting the risk of gastrointestinal disorders, cancers and CVD [85,92,93]. 

Moreover, up to 90% of dietary plant polyphenols [94] including apples, reach the colon intact [48,59,95,96]. The interaction with the gut microbiota is reciprocal, since commensal bacteria transform polyphenols into simple aromatic metabolites while polyphenols have the ability to modulate the gut microbiota composition, inhibiting some bacterial populations and stimulating others [97,98]. Tzounis *et al*. (2011) showed that a high cocoa flavanol drink (494 mg/day) significantly increased fecal bifidobacteria and lactobacilli, but decreased clostridia populations after 4 weeks, compared with a low cocoa flavanol drink (23 mg/day), in a randomized, double-blind, controlled, crossover, human intervention trial of 22 subjects [99]. Similar prebiotic effects have been shown in a study exploring the impact of red wine, dealcoholized red wine and gin consumption on gut microbiota composition in 10 subjects for 20 days [100]. The intake of red wine increased the number of *Enterococcus*, *Bacteroides*, and *Prevotella* genera and decreased the *Clostridium* genera and *Clostridium histolyticum* group [100]. These effects were not observed after the gin consumption, used as an alcohol control, which gave significant increased levels of fecal clostridia and *Bacteroides*, and decreased *Prevotellaceae* [100]. Both red wine and dealcoholized red wine increased the levels of *Blautia coccoides*–*Eubacterium rectale* group, *Bifidobacterium*, *Eggerthella lenta*, and *Bacteroides uniformis*, indicating the beneficial effects of wine polyphenols [100]. Finally, the beneficial observed reductions in total cholesterol (TC) and C-reactive protein (CRP) were related to the changes in bifidobacteria number [100]. However, these beneficial effects on gut health were not confirmed in a recent placebo-controlled, crossover, human intervention study by Wallace *et al*. (2015), where a boysenberry beverage (750 mg polyphenols, anthocyanins and ellagitannis/ellagic acid as the main source) and an apple fiber beverage (7.5 g) were consumed separately and in combination [101]. There was no indication of significant differences in fecal bacteria or SCFA levels after 4 weeks intervention [101]. Differences in polyphenol dose, class and the food matrix is a crucial factor to consider when interpreting these results. The modulation of gut microbiota activity and composition by apple components has been shown in *in vitro* and *in vivo* experiments, the majority in animals and only a few in humans. 

### 3.1. Impact of Apple Components on the Gut Microbiota Composition

#### 3.1.1. *In vitro S*tudies

Pectin is almost completely fermented *in vitro* and thus, can modify the human gut microbiota composition [42,43,44]. Structural characteristics such as DM and molecular weight influence the fermentability properties [31,33,102]. The speed of fermentation and selectivity by the human fecal bacteria for low and high methylated pectins are inconsistent in different studies which may be due to variability in the fecal donor and *in vitro* experimental conditions [31,33,102]. Furthermore it was reported that pectin fermentation was relatively common among *Bacteroides* spp. and only performed by *Eubacterium eligens* among gram-positive anaerobes including species from *Firmicutes* and actinobacteria [103,104]. Later studies show that *Faecalibacterium prausnitzii*, which belongs to the *Firmicutes*, are able to utilize apple pectin and compete with known pectin utilizing species such as *Bacteroides thetaiotaomicron* and *Eubacterium eligens* [105]. However, the fermentability of pectic oligosaccharides (POS) differs from pectin. Comparing the two substrates Olano-Martin *et al.* (2002) showed that POS increased bifidobacteria number whereas pectin increased *Bacteroides* and *Clostridium* [102]. Similarly, it has been shown that apple POS increased bifidobacteria and lactobacilli levels and decreased clostridia and *Bacteroides,* while pectin increased bifidobacteria, clostridia, *Bacteroides* and eubacteria [106]. Thus, these studies indicate that selectivity towards bifidobacteria may only be confirmed for POS, suggesting a prebiotic effect. 

The effects of pure apple PAs on the gut microbiota composition are not well explored. In an *in vitro* batch culture system, inoculated with human faeces, a decrease in SCFA concentration after apple PAs fermentation was reported, suggesting reduction of beneficial saccharolytic fermentation, although, the specific bacteria composition was not determined [107]. It has been shown that PAs possess antimicrobial properties by inhibiting microbial enzymes [108], binding to bacterial cells affecting membrane function [109] and complex formation with metal ions, including iron, affecting bacterial growth [110]. Although there is evidence of a bacteriostatic effect of condensed tannins this inhibition may be selective to particular bacterial species. Animal studies using flavanols from blackcurrant extract, grape pomace or tannin rich diets modified the balance of gut microbiota composition towards beneficial bacteria [111,112,113]. Moreover, it has been suggested that structural differences of the bacterial cell walls and membranes may explain why gram-positive bacteria such as clostridia are more sensitive to bactericidal effects of tannins compared to gram-negative *Prevotella* and *Bacteroides* species [111].

#### 3.1.2. Animal Studies

Short and long term consumption of whole apples and apple components modified the rat caecal microbiota composition [26]. Pectin was considered as the main bioactive component after administration of 7% of apple pectin for 4 weeks by increasing *Clostridium coccoides* populations and the expression of genes encoding the butyryl-coenzyme A (CoA) transferase, which are present in bacteria of the *Clostridium* cluster XIVa, *Roseburia*-*Eubacterium rectale* cluster and *Faecalibacterium prausnitzii*, all known as important butyrate producers [26]. Furthermore, the pectin diet resulted in a decrease of *Bacteroides* spp. and a higher level of butyrate [26]. Although pectin is an important contributor to gut health, exploring the combined effects of apple polyphenols and fiber seems prudent. Moreover, dietary fiber can form a complex with polyphenols referred as “antioxidant dietary fiber” [114] protecting antioxidants which can reach the colon and have local effects in the gut [19]. Apple pomace, a by-product of juice production, and a high source of fiber and polyphenols, has been shown to have beneficial effects by decreasing caecal pH and increasing SCFA in rats compared with a control diet [114]. Similarly, apple pomace extraction juice colloids (5%) increased *Bacteroidaceae*, caecal content weight and SCFA concentration, mainly acetate and propionate, indicating microbial fermentation of pectin [24]. An alcohol apple pomace extract rich in insoluble fiber further increased butyrate concentration and *Eubacterium rectale* cluster compared to the juice colloids and the control [24]. In addition, the same group reported an increase in *Lactobacillus* and *Bifidobacterium* counts after the administration of juice extracted from apple pomace [25]. Moreover, the observed elevated acetate was associated with pectin fermentation whereas the higher total SCFA were linked to polyphenols, including quercetin-3-glucoside [25]. A synergistic interaction of apple pectin and polyphenols have been reported on large intestinal fermentation and lipid metabolism [115], and rat cecum fermentation [116]. In contrast, although an intake of 5% unprocessed apple pomace, containing 61% of fiber and 0.23% of polyphenols increased caecal SCFA compared to a control diet, reducing or removing the polyphenol fraction caused further beneficial effects by increasing the glycolytic activity of caecal microbiota, beneficially modifying the ratio of caecal SCFA and branched-chain fatty acids (BCFA), and decreasing caecal ammonia and colonic pH [117]. Finally, in a recent *ex vivo* study the microbiota balance after *in vitro* fermentation of faeces from diet induced obese mice with nondigestible apple compounds including dietary fiber, extractable and non-extractable phenolics, resembled that in lean controls [118]. Nevertheless, it should be noted that animals metabolize apple components differently from humans [26]. Moreover, the main site of fermentation in rats is the cecum, whereas in humans this occurs in the colon. Thus, human studies are necessary to explore these effects. A summary of the *in vivo* studies is presented in Table 2.

#### 3.1.3. Human Studies

To our knowledge, only two human studies have explored the effects of apples on gut microbiota (Table 2). In a small scale intervention study (*n* = 8), 2 apples per day for 2 weeks significantly increased fecal bifidobacteria, while reducing *Enterobacteriaceae* and lecithinace-positive clostridia, including *C. perfringens* [27]*.* A trend for increased levels of *Lactobacillus, Streptococcus* and *Enterococcus* was also reported [27]. However, this study did not use culture independent microbiology techniques and a control treatment, limiting the ability to accurately assess apple induced changes. In a recent 4 week-study of 23 healthy subjects whole apple and pomace consumption lowered fecal pH and resulted in differences in denaturing gradient gel electrophoresis (DGGE) profile. However, a potential modulation of the gut microbiota population was not confirmed by quantitative PCR [15]. There is a need to further explore the impact of apple consumption on both the composition and metabolic output of the gut microbiota in suitable designed, well powered, controlled, dietary intervention studies using the most up to date culture independent microbiological techniques.

**Table 2:** Effects of apples and apple components on gut microbiota composition and activity.

Type of Study	Duration-Diets-Daily Dose	Techniques Used	Results	Author
Animal (Wistar rats)	6 weeks, 10 rats for each group: Control diet or 5% apple pomace (AP) 1B-juice colloids extract (54.3% soluble and 2.6% insoluble fiber) or 5% AP 4B-juice colloids extract, rich in soluble fiber (78.3% soluble and 1.8% insoluble) or 5% alcohol AP extract, rich in insoluble fiber (22.9% soluble and 73.3% insoluble)	FISH (caecal) Plate count (feaces)	B-juice AP extracts: Total SCFA, acetate and propionate: ↑, pH: ↓ *Bacteroidaceae*: ↑ (faeces) Alcohol AP extract: Total SCFA and butyrate: ↑, pH: ↓ *Bacteroidaceae*: ↑ (faeces) *Eubacterium rectale*: ↑ (caecal)	Sembries *et al.* (2003) [24]
Animal (Wistar rats)	4 weeks, 12 rats for each group: Control diet or Extraction juice from apple pomace	Plate count	Total SCFA and acetate: ↑, pH: ↓ *Lactobacillus*: ↑ *, Bifidobacterium*: ↑ Primary bile acids and neutral sterols: ↑ Secondary bile acids: ↓	Sembries *et al*., 2006 [25]
Animal (Fischer rats)	4 weeks, 8 rats for each group: Control diet or 10 g apple or 7% apple pectin	qPCR	Apple: Butyrate: ↑, pH: *↓*, *Bacteroides spp*: *↓* Apple pectin: Butyrate:↑, pH: *↓*, *Bacteroides spp*: *↓*, *Clostridium coccoides*:↑	Licht *et al.*, 2010 [26]
Animal *ex vivo* (mice)	Granny Smith apple fermented *in vitro* from faeces from diet induced lean (control) and obese mice.	qPCR	*Firmicutes*, *Bacteroidetes*, *Enterococcus*, *Enterobacteriaceae*, *Escherichia coli* and *Bifidobacterium* abundances from obese mice tended to be similar to lean mice after apple fermentation.	Condezo-Hoyos *et al.*, 2014 [118]
Human	2 weeks, 8 subjects: 2 apples	Plate count	Bifidobacteria: ↑ Clostridia: *↓, Enterobacteriaceae*: *↓*	Shinohara *et al.,* 2010 [27]
Human Randomized, single blinded, controlled, crossover	4 weeks, 23 subjects: Control: period of restricted diet or 550 g whole apples or 22 g apple pomace or 500 ml clear apple juice or 500 ml cloudy apple juice	qPCR	No changes in bacteria composition. pH: *↓*	Ravn-Haren *et al.*, 2012 [15]

↑: significant increase; *↓*: significant decrease; SCFA: short chain fatty acids; FISH: fluorescence in situ hybridization; qPCR: quantitative polymerase chain reaction.

### 3.2. Impact of the Gut Microbiota on Apple Components—Focus on Polyphenols

#### 3.2.1. *In vitro*

A study by Deprez *et al.* (2000) was one of the first to show that PA polymers can be degraded by the human colonic microbiota into low molecular weight aromatic acids with different hydroxylation profile and aliphatic side chain length [119]. From the initial ^14^C labelled PAs, 9%–22% found in the metabolite pool and the major microbial metabolites included 3-hydroxyphenylpropionic acid, 3-phenylpropionic acid, 4-hydroxyphenylpropionic acid and 4-hydroxyphenylacetic acid [119]. The ability of the human gut microbiota to ferment apple PAs with different chain length was explored for pure apple, enzymatically digested apple, isolated cell walls, isolated PAs, cell walls-PAs or ciders from Marie Menard and Avrolles apple varieties [107]. The main microbial metabolites included 3-(3,4-dihydroxyphenyl)propionic acid, 3-(3-hydroxyphenyl)propionic acid, 3-phenylpropionic acid and benzoic acid derived from apple flavanols and 2-(3,4-dihydroxyphenyl)acetic acid and 2-(3-hydroxyphenyl)acetic acid from flavonols [107]. The DP of PAs can affect microbial conversions. Increasing the DP might suppress the extension of microbial metabolism and formation of phenolic acids. Moreover, the isolated long chain PAs inhibited the formation of SCFA from cell walls and carbohydrates naturally present in the fecal inoculum which was not noticeable when the PAs were combined with the apple cell walls [107]. Differences in the extent of microbial degradation between monomeric and oligomeric flavanols have been shown in other *in vitro* studies [120,121]. Recently, Ou *et al.* (2014) explored the human microbial metabolism of (−)-epicatechin, (+)-catechin, PC B2 and partially purified apple PCs (Granny Smith variety) [122]. The major microbial metabolites of (−)-epicatechin, (+)-catechin and PC B2 were benzoic acid, 2-phenylacetic acid, 3-phenylpropionic acid, 5-(3′-hydroxyphenyl)-γ-valerolactone and 5-(3′,4′-dihydroxyphenyl)-γ-valerolactone and accounted for over 70% of the total metabolites [122]. Fermentation of all the substrates including apple PCs further produced 2-(3-hydroxyphenyl)acetic acid, 2-(4-hydroxyphenyl)acetic acid, 2-(3,4-dihydroxyphenyl)acetic acid, 3-(3-hydroxyphenyl)propionic acid and hydroxyphenylvaleric acid. Apple PCs produced the lowest amount of metabolites but demonstrated the highest quantity of 3-(3-hydroxyphenyl)propionic acid. Moreover, 2-(3,4-dihydroxyphenyl)acetic acid was only detectable from PC B2 and apple PCs. The lowest recovery of metabolites after 24 h fermentation were observed for the purified apple PCs compared to the other substrates supporting the theory that PCs might slow down the microbiota metabolism [122]. 

Hydroxycinnamic acids that reach the colon may undergo intensive microbial metabolism. Chlorogenic acid has been shown to be converted to 3-(3-hydroxyphenyl)propionic acid and benzoic acid [123] whereas Rechner *et al.* (2004), observed conversion to caffeic acid, 3-(3,4-dihydroxyphenyl)propionic acid, followed by the formation of 3-(3-hydroxyphenyl)propionic acid and finally 3-phenylpropionic acid [124]. Colonic absorption of the last two metabolites can lead to the production of 3-hydroxyhippuric acid and hippuric acid in the liver [124].

Quercetin glycosides may also reach the colon and could serve as a substrate for human gut bacteria. It has been shown that once the quercetin molecule is released by the action of microbial enzymes it can be degraded to 3,4-dihydroxyphenylacetic acid [125]. Phloroglucinol appears as a transient intermediate which might be further degraded to acetate and butyrate [126]. *Eubacterium ramulus* and *Flavonifractor plautii*, among other bacteria are capable of metabolizing quercetin [125,127]. Finally, it is worth mentioning that different fecal donors possess a different intestinal microbiota composition causing a high individual variation and thus, caution is necessary when comparing these *in vitro* results [107].

#### 3.2.2. *In Vivo—*Animal and Human Studies

The urinary metabolome of rats was investigated using an untargeted mass spectrometry based approach after the consumption of 7% apple pectin or 10 g of raw apple compared to a standard diet [128]. The metabolites and potential markers of apple intake that appear in urine included quinic acid, *m*-coumaric, hippuric acid and 3-hydroxy hippuric acid, all related to gut microbial metabolism predominantly of chlorogenic acid. Dihydroxyphenyl-γ-valerolactone an important microbial metabolite of (−)-epicatechin was also identified [128]. The metabolites related to pectin consumption were pyrrole-2-carboxylic acid, 2-furoylglycine and 2-piperidinone. Gut microbial metabolism was also suggested after alterations in plasma metabolite levels in rats fed with fresh apples [129]. 

Animal studies result in different bioconversions compared to humans. For example, although human gut microbiota can metabolize PAs into low molecular weight phenolic acids, these may be poorly metabolized in rats [130,131]. To our knowledge no human study has explored the formation of polyphenol gut microbiota metabolites after apple consumption. However, in a recent study, changes in the blood metabolome after different apple product consumption suggested altered gut microbial metabolism [132]. Studies on cocoa derived products, which are a rich source of oligomeric and polymeric PCs, with similar chemical structures to apples, identified in humans colonic microbial-derived phenolic catabolites, mainly 3-hydroxyphenylpropionic acid, 3,4-dihydroxyphenylacetic acid, 3-hydroxyphenylacetic acid and 3-hydroxybenzoic acid [133]. 

## 4. Cardiovascular Disease Risk

Cardiovascular disease risk factors including, dyslipidemia, endothelial dysfunction and inflammation are some of the biggest health challenges today. Epidemiological evidence suggests that flavonoid consumption may reduce cardiovascular disease risk and apples, as a rich source, may be a major contributor [7,9]. In a Finnish study of 10,054 men and women the link between flavonoid intake and several chronic diseases was explored. Apple intake was strongly inversely correlated with ischemic heart disease mortality, thrombotic stroke and total mortality [7]. Apples were also associated with a reduced risk of coronary heart disease and total cardiovascular disease mortality, as shown in the Iowa Women’s Health Study, where 34,489 subjects free of CVD were followed up for 16 years [9]. In contrast, a prospective study of 38,445 women [134] and a 5 year follow up of 805 elderly men [135] found only non-significant inverse associations between CVD risk and apple consumption.

### 4.1. Lipid Metabolism

#### 4.1.1. Animal Studies

The effects of whole apples or specific apple constituents on lipid metabolism have been investigated in a number of animal studies (Table 3). Fiber, mainly pectin, was considered initially as the main apple component responsible for the cholesterol lowering properties [136,137,138] and as shown by Sanchez *et al.* (2008) to a similar extent as oat β-glucan which is known for its hypocholesterolemic effects [35]. Apple polyphenols can also play an important role. Unripe apples, rich in PCs, significantly decreased serum and liver cholesterol, as well as atherogenic indices, after a hypercholesterolemic diet [139]. Polyphenols and in particular catechin, epicatechin and PC B1 were considered responsible for the lipid lowering effects of a portuguese apple variety (Bravo de Esmolfe) in hypercholesterolemic rats [140]. Other studies indicated that apple polyphenols could compensate the dyslipidemic effects of a high cholesterol diet [20,140,141,142,143]. However, the combined effect of apple fiber and polyphenol proved more efficient in reducing plasma cholesterol levels [115], and hepatic cholesterol [144] than the individual components. An apple diet consisting of 10% or 15% lyophilized apple improved lipid metabolism and significantly decreased TC respectively [145,146]. Increasing the amount of lyophilized apple to 20%, lowered plasma cholesterol, low density lipoprotein-cholesterol (LDL-C), high density lipoprotein cholesterol (HDL-C) and triacylglycerol (TAG) concentrations in liver and heart in hypercholesterolemic genetically obese Zucker rats compared to a control diet [147]. Apple pomace, reduced TC, TAG and serum atherogenic index [117]. The importance of apple pomace on lipid metabolism was also demonstrated in a more recent study [148] whereas Sembries *et al.*, (2004) did not find effects on serum lipid levels [149]. Finally, apple peel had a higher positive effect on plasma lipids compared to the pulp which was attributed to the higher content of bioactive components in the peel [150].

#### 4.1.2. Human Studies

Apple pectin has been shown to decrease plasma TC in humans [151]. A meta-analysis concluded that 1 g of pectin could decrease TC and LDL-C by 0.070 and 0.055 mmol/L respectively [37]. The combined effect of apple fiber and gum arabic supplemented in an apple juice decreased TC (8%) and LDL-C (14%) in mildly hypercholesterolemic men compared to the unsupplemented apple juice [152]. Pure apple polyphenols can also decrease TC and LDL-C in a dose-depended manner as was shown in mild hypercholesterolemic subjects [11]. Although the main effects were observed after the highest concentration (1500 mg), which is considerably higher with the amount found in an apple, a later study by the same group found similar effects with 600 mg, together with a decrease in visceral fat [12]. In contrast, a cloudy apple juice containing 800 mg of polyphenols had no effects on blood lipids but a significant interaction between IL-6-174 G/C polymorphism and body fat reduction [13]. It was identified in an early study that the consumption of 3 apples per day for 4 months significantly reduced TC and increased HDL-C in 76 mildly hypercholesterolemic subjects compared to a control group (less than 3 apples per week) [153]. The effects of dried apples were explored in a long intervention parallel study (12 months) of 160 postmenopausal women compared to dried plum (comparative control) [14]. TC concentration was significantly decreased at 6 months compared to the control, and at 3 months within the intervention group. More recently, 23 healthy subjects consumed whole apples (550 g), apple pomace (22 g), clear and cloudy apple juices (500 ml) daily, each for 4 weeks including a control period [15]. Compared to the control period only, trends were observed for a reduction in LDL-C after whole apple (6.7%), pomace (7.9%) and cloudy apple juice (2.2%) intake [15]. 

**Table 3:** Effects of apples and apple components on lipid metabolism in animals.

Animal Type- Number (*n*)- Study Duration-Design	Diets-Daily Dose	Results	Author
Wistar rats *n* = 12 each group 3 weeks/parallel	Control diet or 15% lyophilized apple	TC: ↓ 9.3% Faecal TS excretion: ↑ Faecal TC excretion: ↑	Aprikian *et al.*, 2001 [145]
Wistar rats *n* = 10 each group 40 days/parallel	Control cholesterol (3g/kg) diet or 10% apple pomace fiber	TC: ↓ 18.4% LDL-C: ↓ 31.2% HDL phospholipids: ↑ 19% TAG: ↓ 14.8% Liver TC: ↓ 26.3%	Leontowicz *et al.*, 2001[138]
Lean (Fa/-) and obese (fa/fa) Zucker rats *n* = 8 each group 3 weeks/parallel	Control diet or 20% lyophilized apple	TC: ↓ 22% (Obese group) LDL-C: ↓ 70% (Obese group) HDL-C: ↓ 26% (Obese group) Liver and heart TAG: ↓ (Obese group) Faecal BA excretion: ↑ (Lean group)	Aprikian *et al.*, 2002 [147]
Wistar rats *n* = 8 each group 4 weeks/parallel	Control cholesterol (1%) diet or 10% whole dry apples	TC: ↓ 20% LDL-C: ↓ 32.6% TAG: ↓ 17% Liver TC: ↓ 29.6%	Leontowicz *et al.*, 2002[146]
Wistar mild hypercholesteolemic rats *n* = 10 each group 3 weeks/parallel	Control diet or Freeze dried pectin, 5% (PEC) or High polyphenol cider apple extract, 10% (PL) or Mixed diet: PEC + PL	TC: ↓ 24% (PEC + PL) TAG: ↓ 29% (PL) ↓ 35% (PEC + PL) Liver TC and TAG: ↓ (PEC and PEC + PL) Faecal BA excretion: ↓ (PEC and PEC + PL) Faecal NS excretion: ↑ (PEC and PEC + PL)	Aprikian *et al.*, 2003 [115]
Wistar rats *n* = 8 each group 4 weeks/parallel	Control cholesterol (1%) diet or apple peel, 10% (Apeel) or apple pulp, 10% (Apulp)	TC: ↓ 21.6% (Apeel), ↓ 19.4% (Apulp) LDL-C: ↓ 35.3% (Apeel), ↓ 33.3% (Apulp) TAG: ↓ 18% (Apeel), ↓ 14.6% (Apulp) Liver TC: ↓ 31.6% (Apeel), ↓ 27% (Apulp)	Leontowicz *et al.*, 2003[150]
Sprague-Dawley rats *n* = 8 or 9 each group 30 days/parallel	Control cholesterol (0.5%) diet or 0.2% apple polyphenols rich in oligomeric procyanidins (AP) or 0.5% AP or1% AP	TC: ↓ (all treatments) HDL-C: ↑ (1% AP), ↓ (0.2% AP) HDL-C/TC: ↑ (0.5% and 1% AP) Liver TC: ↓ (0.5% and 1% AP) Atherogenic indices: ↓ (0.5% and 1% AP) Faecal acidic and neutral steroid excretion: ↑ (0.5% and 1% AP)	Osada *et al.*, 2006 [139]
ApoE deficient mice (apoE-KO) *n* = 16 each group 4 months/parallel	Control diet or apple polyphenols (AP), equivalent to 1.6 g/day for humans orapple fiber (AF), equivalent to 50 g/day for humans or Mixed diet: AP + AF	Liver TC: ↓ 22% (AP + AF) Atherosclerotic lesions: ↓ (all treatments)	Auclair *et al.*, 2008 [144]
Hypercholesterolemic hamsters *n* = 8 each group 12 weeks/parallel	Control atherogenic diet orapple (A) or apple juice (AJ) Equivalent to daily consumption of 600 g apples or 500 ml of juice for humans	TC: ↓ 11% (A) ↓ 24% (AJ) Non HDL-C: ↓ 30% (A) ↓ 55% (AJ) TC/HDL-C: ↓ 25% (A) ↓ 38% (AJ) Aortic fatty streak area: ↓ 48% (A) and 60% (AJ)	Decorde *et al*., 2008 [142]
Golden Syrian hamsters *n* = 13 each group 6 weeks/parallel	Control atherogenic diet, 0.1% cholesterol or 0.3% apple polyphenols (AP) or 0.6% AP	HDL-C: ↑14.7% (0.3% AP) ↑16.5% (0.6% AP) Non HDL-C: ↓ 20% (0.3% AP) ↓ 36.7% (0.6% AP) TAG: ↓ 31.9% (0.6% AP) Faecal BA excretion: ↑ (0.3% and 0.6% AP) Faecal NS excretion: ↓ (0.3% and 0.6% AP)	Lam *et al.*, 2008 [143]
Zucker fatty rats *n* = 10 each group 15 weeks/parallel	Control diet or High methoxylated apple pectin, 10% (HMAP) or β-glucan, 10%	TC: ↓ (HMAP and β-glucan) HDL-C: ↑ (HMAP) Non HDL-C: ↓ (HMAP and β-glucan) TAG: ↓ (HMAP and β-glucan, higher effect for HMAP)	Sanchez *et al.*, 2008 [35]
Rabbits *n* = 8 each group 2 months/parallel	Control cholesterol (1%) diet or 5 ml apple juice (low dose, LD) or 10 ml apple juice (high dose, HD)	TC: ↓ 75% (LD), ↓ 77% (HD) LDL-C: ↓ 70% (HD) HDL-C: ↑ 86% (HD) TAG: ↓ 61% (LD), ↓ 59% (HD) Atherosclerotic lesions: ↓ (LD and HD)	Setorki *et al.*, 2009 [20]
Wistar rats *n* = 8 each group 4 weeks/parallel	Control diet or 5% AP: apple pomace (61% dietary fiber (DF), 0.23% polyphenols (PP)) or 5% APE: ethanol extracted apple pomace (66% DF, 0.1% PP) or 5% APA: ethanol/acetone extracted apple pomace (67% DF, 0.01% PP)	TC: ↓ 19% (AP and APA) TAG: ↓ 26% (APA) ↓ 38% (AP) Atherogenic index, log TAG/HDL-C: ↓ (AP)	Kosmala *et al.*, 2011 [117]
Wistar hypercholesterolemic rats *n* = 8 each group 30 days/parallel	Control cholesterol (2%) diet 20% apples from 3 different varieties: - Golden (G) - Malapio da serra (MS) - Bravo de esmolfe (BE) (highest polyphenol and antioxidant capacity)	TC: ↓ 21% (BE) LDL-C: ↓ 20.4% (BE) TAG: ↓ 27.2% (BE)	Serra *et al.*, 2012 [140]
Sprague–Dawley rats *n* = 8 each group 5 weeks/parallel	Control high fat diet or 10% apple pomace (AP) or 10% apple juice concentrate (AC)	TC: ↓ 23% (AP), ↓ 22% (AC) LDL-C: ↓ 34% (AP), ↓ 32% (AC) HDL-C: ↑ 12% (AP) HDL-C/TC: ↑ (AP and AC) TAG: ↓ 30% (AP), ↓ 27% (AC) Liver TC: ↓19% (AP) Liver TAG: ↓ 21% (AP), ↓ 10% (AC) Atherogenic index: ↓ (AP and AC)	Cho *et al.*, 2013 [148]

↑: significant increase compared to the control diet; *↓*: significant decrease compared to the control diet; TC: total cholesterol; LDL-C: low density lipoprotein-cholesterol; HDL: high density lipoprotein; TAG: triacylglycerols; TS: total steroids; NS: neutral sterols; BA: bile acids.

In contrast, some studies concluded that apples or apple components did not show any significant beneficial effects with a limited number suggesting some adverse effects. The lipid lowering properties of gum arabic-pectin supplementation was not observed in a study of 85 hypercholesterolemic subjects [154]. Consumption of clear apple juice resulted in a significantly higher level of TC and LDL-C compared to whole apple or apple pomace [15]. Blood lipid levels did not change after the consumption of fresh whole apples [155,156] or apple juice [155]. Apple consumption increased TAG levels in hypercholesterolemic overweight women in Brazil after a period of 12 weeks [16]. However, a small but significant weight loss of 1.22 kg, was also reported after the fruit consumption, suggesting beneficial modification of the energy intake and satiety [16]. Similarly, a control group (no apple intake) of overweight men decreased serum TAG and very low density lipoprotein compared to the intervention apple group [157]. The most recent human intervention studies are presented in Table 4. 

In general, the majority of these human studies provide some evidence to support beneficial effects on lipid metabolism, mainly TC reduction. In a mini-review it was reported that 3 apples per day could reduce cholesterol by 5%–8% (approximately 0.5 mmol/L) [158]. The specific amount and type of polyphenols and fiber that are responsible for these effects requires exploration. The delivery matrix of these bioactive components is also crucial. Beneficial effects are shown with fresh or dry apples and cloudy apple juice, however clear apple juice has been associated with adverse effects. Apple pomace is also a valuable material since it contains an important fraction of the polyphenols and fiber of the whole apple. More large scale randomized and well controlled human intervention studies are required to confirm these effects and to explore the potential mechanisms using realistic doses that can be successfully incorporated to a habitual human diet.

#### 4.1.3. Potential Lipid-Lowering Mechanisms

##### Modulation of Bile Acid Enterohepatic Circulation 

Bile acids (BAs) are known to play an important role in lipid metabolism. BA synthesis is performed in the liver and regulated mainly by cholesterol 7α-hydroxylase (CYP7A1), an enzyme that converts cholesterol to cholic acid (CA) and chenodeoxycholic acid (CDCA) as the primary forms [159]. Prior to their secretion via the gall bladder, BAs are conjugated with the amino acids taurine or glycine to form bile salts. After a meal bile salts enter the duodenum, facilitating the metabolism and the absorption of dietary lipids. Most bile salts are then reabsorbed in the distal ileum. However, a small percentage escape reabsorption and are deconjugated by gut bacteria. These are then converted into secondary bile acids, which are either excreted in the faeces or absorbed and return back to the liver where together with the absorbed primary bile acids and salts are reconjugated and resecreted completing one cycle of the enterohepatic circulation [159]. The enzyme responsible for the deconjugation of bile salts is bile salt hydrolase (BSH), which has been isolated from several gut bacteria including species of bifidobacteria and lactobacilli [160]. Once deconjugated, bile acids are less soluble leading to reduced reabsorption and increased faeces excretion [161]. Consequently more cholesterol is removed from the circulation for *de novo* synthesis [161]. 

**Table 4:** Effects of apples and apple components on blood lipid levels in humans.

Subjects-Study Duration-Design	Diets-Daily Dose	Results	Author
25 healthy men/women 6 weeks Randomized, crossover	340 g apple or 375 ml apple juice	No changes: TC, LDL-C, HDL-C and TAG	Hyson *et al.*, 2000 [155]
49 hypercholesterolemic, overweight women 12 weeks (35 women) Randomized, parallel	300 g apple or 300 g pear or 60 g oat cookies	TC: ↓ (oat group) TAG: ↑ (fruit group)	de Oliveira *et al.*, 2003[16]
48 hypercholesterolemic men/women 4 weeks Randomized, double-blinded, placebo-controlled, parallel	Control: supplement without polyphenols or Low dose: 300 mg apple polyphenols (AP) or Medium dose: 600 mg AP or High dose: 1500 mg AP	TC: ↓ 4.5% (from baseline for High dose) LDL-C: ↓ 7.8% (from baseline for High dose) No changes: HDL-C and TAG	Nagasako-Akazome *et al.*, 2005 [11]
15 elderly 4 weeks	Fresh apples (2 g/kg body weight, approximately 1 apple)	No changes: TC, LDL-C, HDL-C and TAG	Avci *et al.,* 2007 [156]
48 moderately obese men/women 12 weeks Randomized, double-blinded, placebo-controlled, parallel	Control: capsules without polyphenols or 600 mg apple polyphenols capsules	TC: *↓* (from baseline and control group) LDL-C: *↓* (from baseline) No changes: HDL-C and TAG VFA: *↓* (from control group) Adiponectin: ↑ (from control group)	Nagasako-Akazome *et al.*, 2007 [12]
46 overweight, hyperlipidemic men 8 weeks Randomized, controlled, parallel	Control: no apple intake or 300 g apple	No changes: TC, LDL-C and HDL-C TAG: *↓* (in control group compared with the apple group) VLDL-C: *↓* (in control group compared with the apple group)	Vafa *et al.*, 2011 [157]
68 overweight men 4 weeks Randomized, blinded, placebo-controlled, parallel	Control: beverage without polyphenols or 750 ml cloudy apple juice	No changes: TC, LDL-C, HDL-C and TAG % total body fat: ↓ (from control group) Body fat mass: ↓ (only in IL-6-174 C/C variant compared with G-allele carriers).	Barth *et al.*, 2012 [13]
160 postmenopausal women 1 year Randomized, single blinded, controlled, parallel	Dried plum (comparative control) or 75 g dried apples	TC: ↓ (from control group) TC: ↓ 13% (from baseline) LDL-C: ↓ 24% (from baseline) TC:HDL-C: ↓ (from baseline) LDL:HDL-C: ↓ (from baseline) No changes: HDL-C and TAG	Chai *et al.*, 2012 [14]
23 healthy men/women 4 weeks Randomised, single blinded, controlled, crossover	Control: period of restricted diet or 550 g whole apples (WA) or 22 g apple pomace (AP) or 500 ml clear apple juice (AJ) or 500 ml cloudy AJ	Treatment resulted in significant effects in TC and LDL-C. Clear AJ: ↑ 5% TC, ↑ 6.9% LDL-C (compared with WA and AP) No changes: HDL-C and TAG	Ravn-Haren *et al*, 2012 [15]
20 healthy young men/women 4 weeks Randomized, crossover	500 ml of two cloudy apple juices: 510 mg/L catechin equivalent and 60 mg/L vitamin C (VCR) or 993 mg/L catechin equivalent and 22 mg/L vitamin C (PR)	TC: ↓ 4% (VCR) No changes: LDL-C, HDL-C and TAG	Soriano-Maldonado *et al.*, 2014 [162]

↑: significant increase; ↓: significant decrease; TC: total cholesterol; LDL-C: low density lipoprotein cholesterol; VLDL-C: very low density lipoprotein cholesterol; HDL-C: high density lipoprotein cholesterol; TAG: triacylglycerols; VFA: viscelar fat area.

Animal studies showed that apple polyphenol supplementation increased the excretion of bile acids in faeces [139,143]. This may be related to binding of polyphenols with bile acids enhancing their excretion in the faeces (Table 2). Apple polyphenols and mainly PCs may directly enhance the activity of CYP7A1, the first and rate-limiting enzyme in bile acid synthesis [139]. Moreover, it has been suggested that catechins and PCs could inhibit intestinal cholesterol absorption by affecting micellar solubility and may also increase direct excretion of cholesterol and lipids in faeces [163,164]. 

Apple pectin as a soluble, nondigestible and viscous fiber has the ability to increase bile acids and/or cholesterol passage to the colon and faeces through formation of complexes and may also interrupt the enterohepatic circulation and reduce plasma cholesterol levels [136,137,147,149]. In contrast a decreased faecal BA and increased neutral sterol excretion after intake of apple pectin (alone or combined with apple polyphenols) suggests cholesterol lowering properties in parallel with a high rate of bile acid reabsorption [115]. Further studies are necessary to explore this discrepancy and further assess the complete bile acid pool in circulation. 

Additionally, intestinal microbiota may play a role in conversion of cholesterol to coprostanol which is subsequently excreted in faeces [165]. Deconjugated BAs can also act as signalling molecules affecting activation of the farnesoid X receptor (FXR), a nuclear receptor that inhibits hepatic *de novo* lipogenesis and is responsible for other physiological processes which can impact on cholesterol catabolism and lipid absorption from the intestine [166,167,168,169]. 

##### Modification of Lipid Metabolism 

Apple polyphenols may affect lipid metabolism through several other mechanisms including, activation of fatty acid β-oxidation and cholesterol catabolism in the liver [139,164,170], inhibition of hepatic fatty acid synthesis [141,164], decreasing cholesterol esterification and secretion of apoB-containing lipoproteins [171] and suppression of cholesteryl ester transport protein (CETP) activity improving the distribution of cholesterol in lipoproteins [143]. Moreover, apple polyphenols have been associated with a reduction in total body weight gain [24], adipose tissue weight [141,148,172], visceral fat [12,164] and leptin levels [164]. PAs can inhibit the action of digestive enzymes such as lipase and amylase with beneficial effects on lipid and glucose metabolism. Oligomeric apple PCs have been shown to inhibit pancreatic lipase activity, with increased inhibition associated with a high degree of polymerization, affecting postprandial TAG absorption [173]. Upregulation of lipoprotein lipase (LPL) activity has been suggested as an alternative mechanism of TAG lowering [174]. PAs may also regulate lipid metabolism by activating FXR and by modulating other nuclear receptors such as small heterodimer partner (SHP) and peroxisome proliferator-activated receptors (PPARs) as well as transcription factors like steroid response element binding protein 1 (SREBP1) [175,176,177]. In addition, apple phenolic compounds have been associated with a reduction in LDL oxidation, an important contributor to atherosclerosis, as has been shown in *in vitro* [178,179,180] or in animal models [140,147,181].

Pectin has a major role in cholesterol lowering by inhibiting cholesterol absorption and uptake, influencing micelle formation and affecting transit time [35,145]. The origin and physicochemical properties of pectin, including molecular weight, DM and viscosity affect the efficacy of the mechanisms [34,35,37,136,137,182]. For example, by increasing the DM a greater proportion of bile acids are transported to the distal colon [183] increasing faecal excretion [149,183]. Production of SCFA can independently affect these activities. Butyrate plays a major role in colonic function, in addition it has been shown to inhibit liver cholesterol synthesis, whereas acetate and propionate have an impact on metabolic processes at a systemic level, and may possess opposing effects on lipid metabolism [78]. While propionate may inhibit cholesterol synthesis, acetate could increase hepatic lipogenesis, however, the results are inconsistent [78]. Finally, the potential lipid lowering effects of apples are probably due to the combined/synergistic effects of polyphenols and fiber rather than the individual components. Human intervention studies are necessary to support these mechanisms.

### 4.2. Vascular Function and Blood Pressure

Endothelial dysfunction is considered as an early marker in the pathogenesis of atherosclerosis and its complications [184]. Thus, endothelial function can serve as an indication for cardiovascular health. Nitric Oxide (NO) is an important endothelium-derived vasodilator produced from its precursor L-arginine via the enzymatic action of endothelial NO synthase (eNOS) [185]. A defect in the NO production is considered as the main mechanism of endothelial dysfunction and thus, impaired endothelium dependent vasodilation. Flavonoids have been shown to increase NO status, yet few human studies have explored the potential role of apple polyphenols on vascular function and blood pressure. Higher flow mediated dilation (FMD) of the brachial artery, increased NO status and lowered systolic blood pressure was observed in 30 healthy subjects after the acute intake of an apple blend providing 184 mg of quercetin and 180 mg of (−)-epicatechin [17]. Flavonoids can increase NO by stimulating eNOS activity, protect NO from free radicals and inhibit the synthesis of vasoconstrictor endothelin-1 [186,187]. Similarly, both a low and a high apple puree intake (230 g) containing 25 and 100 mg of epicatechin, respectively, increased plasma NO metabolite levels (at 6 h) and attenuated platelet reactivity (at 2 and 6 h) acutely in a study of 25 healthy subjects, but showed no effects after the daily consumption for 2 weeks [18]. No effect was reported in vascular function (assessed by FMD) after a 4-week cross over intervention with 40 g of lyophilized polyphenol rich apples, in 30 hypercholesterolemic subjects [188]. Differences in the study design, food matrix, and polyphenol composition may account for the different results. Thus, an improvement in vascular function with dietary flavonoids has been shown mainly in acute studies with flavanol monomers, mainly (−)-epicatechin, considered responsible for these effects [189]. In the chronic study these monomers were not present in blood after a 10-hour fast which could explain the lack of effects. Although PAs microbial metabolites were present in the blood these had no apparent effect. This has been supported by a study claiming that gut microbial metabolites from PCs may not be responsible for the vascular effects [190]. However, other chronic studies providing PC rich sources such as chocolate and tea have shown beneficial effects and increased FMD (Hooper *et al*., 2008). Moreover, it has been shown *in vitro* that oligomeric PCs from apples, cocoa, red wine and cranberries were responsible for the inhibition of endothelin-1 mainly between the tetramer to heptamer range whereas monomers lacked any activity [191,192]. Similarly, large oligomers from grape seed and cocoa were responsible for endothelium-dependent vasodilation effects [192,193,194]. In addition, microbial polyphenol metabolites, that also appear after apple intake, including benzoic acids, cinnamic acids, chlorogenic acid and 3-(3,4-dihydroxyphenyl)propionic acid have been shown to inhibit platelet activation and aggregation [195,196,197], angiotensin converting enzyme (ACE) action [198] and increase eNOS expression [199]. Other studies have mainly focused on quercetin, a flavonol widely found in apples, and the potential hypotensive effects, as reviewed by Larson *et al.* [200]. A daily administration of 730 mg of quercetin significantly decreased blood pressure in hypertensive, but not in prehypertensive subjects after 28 days [201]. No change in blood pressure was reported with 1000 mg quercetin and 200 mg quercetin rhamnoglucoside in 27 normotensive subjects [202]. In contrast, a lower quercetin dose (150 mg) reduced blood pressure in subjects homozygous for the apolipoprotein E3 genotype only [203]. Apple peel has been shown to inhibit ACE activity *in vitro*, with quercetin-3-*O*-glucoside and the metabolite quercetin-3-*O*-glucuronic acid the most effective, suggesting antihypertensive properties [204]. Apples contain approximately 5 mg of quercetin per 100 g and thus any potential hypotensive effect may be due to synergistic interaction of the other polyphenols. Further chronic human studies are necessary to assess the impact of apple product consumption on vascular function and blood pressure.

### 4.3. Inflammation

Inflammation plays a key role in the pathology of atherosclerosis and coronary heart disease. There is a strong evidence base that polyphenols can exert anti-inflammatory/immunomodulatory activities [205,206], however, few studies have focused on the effects of apple polyphenols. The potential anti-inflammatory effect of apple PCs from 109 different cultivars was tested using cell-based assays [207]. Cultivars with a high PC content were able to inhibit nuclear factor-kappa B (NF-κB), a transcription factor involved in the induction of pro-inflammatory enzymes including cyclooxygenase-2 (COX-2) and the inducible nitric oxide synthase (iNOS), as well as the expression of inflammation related-genes such as those for tumor necrosis factor-alpha (TNF-α), interleukin-1β (IL-1β), IL-6 and IL-8 [207]. Jung *et al.* (2009) reported that PC B1, PC B2 and phloretin from apple juice extracts were related to an anti-inflammatory activity *in vitro* [208]. In an aninal study a high cholesterol diet supplemented with apple juice significantly decreased CRP levels in rabbits [20]. The administration of 7.6% lyophilized apple, rich in polyphenols, in particular PAs and hydroxycinnamic acids (Marie Menard cider variety), for 3 months, reduced colonic inflammation compared to a low polyphenol intake (Golden Delicious) in HLA-B27 transgenic rats, which develop spontaneous intestinal inflammation [19]. Interestingly, the high polyphenol variety had a dramatic impact on *Bacteroides fragilis*, a group of bacteria thought to be involved in the etiology or maintenance of inflammatory bowel disease (IBD). Inhibition of the NF-kB transcription factor, suggested as a potential mechanism [19] [207] has also been reported in other human cell studies with apple extracts [209,210]. The beneficial effect of Marie Menard apples was further confirmed in rats showing decreased colonic and systemic inflammation [22]. Furthermore apple pectin has been associated with anti-inflammatory effects in animal studies by down regulating pro-inflammatory cytokine expression and immunoglobulin production in the colon [211] and systematically by reducing plasma TNF-α [21]. Citrus pectin has been reported to inhibit iNOS and COX-2 expressions in lipopolysaccharide (LPS)-activated macrophages, with greater inhibition from the pectin with the higher DM [212]. Studies in humans are scarce. In an epidemiological study of 8335 US subjects the intake of apples was inversely associated with serum CRP levels [10]. Whereas an intervention study of 77 subjects showed no association between apple consumption and anti-inflammatory activity [213]. 

Gut microbiota composition may also modulate systemic inflammation. LPS, a constituent of gram-negative bacteria triggers the secretion of pro-inflammatory molecules. Elevated levels of LPS in blood circulation, mainly after a high fat diet contribute to metabolic endotoxemia which plays a major role in the pathophysiology of the metabolic syndrome and the progression of atherosclerosis [214,215,216]. Prebiotic dietary fiber, pectin and apple polyphenols through the potential modification of the intestinal microbiota may reduce metabolic endotoxemia by improving gut barrier function and reducing intestinal permeability and uptake of LPS [214,217,218,219]. In high fat fed mice, prebiotic oligofructose reduced endotoxemia and inflammation by increasing bifidobacteria levels [214]. Microbiota-derived polyphenol metabolites may also be responsible for these anti-inflammatory effects. It has been reported that quercetin can exert anti-inflammatory effects in rats, only when it is released from its glycosylated forms (e.g., quercetin 3-rhamnoside) by the action of the intestinal microbiota. [220]. Other *in vitro* and *in vivo* experiments have indicated that polyphenols found in apples may reduce intestinal inflammation in humans after microbial degradation suggesting beneficial effects, locally, at the intestinal level. [221]. Finally systemic effects have been reported in humans. Dihydroxylated phenolic acids, derived from the microbial degradation of PAs, mainly 3,4-dihydroxyphenylpropionic acid and 3,4-dihydroxyphenylacetic acid, significantly inhibited the secretion of pro-inflammatory cytokines including, TNF-α, IL-1β, and IL-6 in LPS stimulated peripheral blood mononuclear cells from 6 healthy volunteers [222].

## 5. Conclusions

There is some supporting evidence to suggest that apples and apple components may beneficially modulate CVD risk factors. The strongest effects are related to lipid metabolism where evidence shows frequent apple consumption can reduce TC. Few studies have focused on vascular function and evidence for an anti-inflammatory activity is also limited and derived mainly from *in vitro* and animal studies. Microbiota-derived metabolites exert various biological activities and could contribute to the beneficial effects observed. A potential prebiotic impact of apple ingestion may be an important mechanism of CVD risk marker reduction. An increasing body of evidence highlights the important regulatory role mediated by microbe:host co-metabolic processes, especially bile acid metabolism. Both polyphenols, especially complex polyphenols such as PAs and dietary fiber, can influence the enterohepatic circulation by binding bile acids in the intestine, in a manner similar to pharmaceutical bile acid chelating agents, or by changing the profile of gut bacteria, modifying their potential to deconjugate and hydrolyse bile acids into secondary bile acids. Both the quantity and relative chemical profiles of bile acids returning to the liver will determine the regulatory potential of these important cell signaling molecules. Moreover, dietary polyphenols may directly play a cell signaling role by modulating transcription factors that regulate important physiological functions, such as intestinal permeability, fat absorption, bile acid metabolism, hepatic lipid/cholesterol metabolism, glucose homeostasis and systemic inflammation. However, many of the mechanisms that impact on physiological processes related to CVD are in animal and *in vitro* models and remain to be convincingly demonstrated in human studies. More suitable powered, randomized, controlled, long term, human dietary intervention studies using metagenomic and metabolomic techniques are required to progress this research area.
